# Systematic Review: Culturally Tailored Digital Substance Use Prevention Interventions for Black Adolescents

**DOI:** 10.1016/j.jaacop.2026.03.007

**Published:** 2026-04-02

**Authors:** Kammarauche Aneni, Destiny Pegram, Jenny Meyer, Melissa C. Funaro, Roberta L. Bruhn, Uzochukwu Imo, Aaron Hogue, José Szapocznik, Ijeoma Opara

**Affiliations:** a Yale University, New Haven, Connecticut; b University of Ibadan, Ibadan, Nigeria; c Partnership to End Addiction, New York, New York; d University of Miami, Miami, Florida

**Keywords:** Black adolescents, culturally tailored interventions, digital interventions, substance use prevention

## Abstract

**Objective:**

Black adolescents differ from adolescents of other racial/ethnic groups in prevalence, patterns, risk and protective factors, and consequences of substance use, underscoring the need for culturally tailored interventions. Black adolescents also experience unique barriers in access to interventions that may be amenable to digital interventions. However, based on available literature, no systematic review has examined the efficacy of culturally tailored digital interventions for substance use prevention among Black adolescents. Here, existing culturally tailored digital interventions for substance use prevention among Black adolescents were systematically reviewed, and their efficacy in preventing substance use was examined.

**Method:**

A systematic search of biomedical research databases was conducted. Search terms included controlled vocabulary terms and free text terms for concepts of digital interventions, substance use, and clinical trials. Randomized controlled trials comparing a culturally tailored intervention for Black adolescents with a control intervention were included. The level of cultural tailoring (surface- vs deep-level tailoring) was determined, and the impact of interventions on substance use outcomes was assessed. The quality of studies was evaluated using the National Institutes of Health tool for quality assessment of controlled intervention studies. A narrative synthesis summarizing results by study characteristics, intervention characteristics, and study outcomes/findings was also conducted.

**Results:**

Eleven articles comprising 6 studies testing 6 interventions met inclusion criteria. The proportion of Black adolescents varied among studies from 41% to 100% (n = 109-421). Four interventions significantly improved overall substance use outcomes with effect sizes ranging from 0.002 to 1.5 for alcohol use, 0.11 to 1.57 for marijuana use, 0.002 to 0.64 for tobacco use, and 0.009 to 0.35 for other drugs. Five interventions also improved antecedents to substance use (eg, self-efficacy, intentions to use drugs, parental monitoring). Effects persisted at long-term follow-up (≥12 months) among 75% of interventions that examined these outcomes. Cultural tailoring varied widely among interventions, with 4 studies reporting surface-level adaptations, 1 study reporting deep-level adaptations, and 1 study reporting both surface- and deep-level adaptations. Cultural tailoring largely focused on intervention content, with only 2 studies describing additional tailoring at the implementation level. One study assessed the impact of cultural tailoring on intervention outcomes. Interventions were tested in various settings, including community (2 studies), home (1 study), school (1 study), emergency department (1 study), and primary care (1 study); were delivered using a CD-ROM/computer (5 studies) or videotape (1 study); and comprised different types of interventions, including universal (4 studies), selective (1 study), and indicated (1 study).

**Conclusion:**

Although the number of available studies is limited, culturally tailored digital interventions appear to be effective at improving substance use outcomes among Black adolescents and have been tested across various settings. However, the impact of cultural tailoring is challenging to isolate, given the wide variability in the description of culturally tailored content, the lack of homogeneity in the study samples, and the lack of evaluation of the effect of cultural tailoring on intervention outcomes. Given rapid advances in digital technologies and the pressing need to curtail the rise in substance use among Black adolescents, it is imperative to design, test, and implement digital interventions that meet the needs of Black adolescents.

**Study registration information:**

Culturally adapted digital substance use interventions for black adolescents: a systematic review; https://www.crd.york.ac.uk/PROSPERO/view/CRD42023452522

## Plain language summary

This systematic review examined studies that tested whether digital interventions (eg, online, computer, or mobile phone apps) designed for Black adolescents were effective in improving substance use outcomes. It also examined ways in which each of these interventions were tailored for Black adolescents. Despite the limited number of available studies, the results show that culturally tailored digital interventions may be effective at improving substance use outcomes in Black adolescents across various settings.

Black adolescents in the United States differ from adolescents of other racial/ethnic groups in prevalence,^1^ initiation,^2^ patterns,^3^, ^4^, ^5^ risk and protective factors,^6^, ^7^, ^8^, ^9^ and consequences of substance use,^10^, ^11^, ^12^, ^13^ underscoring the need for culturally tailored substance use interventions. For example, Black adolescents ages 12 to 17 years have higher rates of cannabis use and lower rates of alcohol and tobacco use compared with white adolescents of similar age.^1^^,^^3^^,^^14^ In addition to differences in drug use, Black adolescents often struggle with increased physical, social, and legal consequences due to substance use. The most recently available data from 2019-2020 reveal that Black youth ages 15 to 24 years had the largest increase in drug overdose rates: 86% vs 51% and 34% for Hispanic and non-Hispanic White peers, respectively.^15^ Black adolescents are more likely to progress to substance use disorders,^16^^,^^17^ and Black adults are at increased risk of mortality from drug use.^10^, ^11^, ^12^ Black adolescents are at increased risk of drug-related arrests^13^ and are less likely to complete high school following a drug-related arrest^18^ compared with non-Hispanic White adolescents.

Black adolescents also have decreased access to mental health and substance use prevention interventions and treatment services.^19^, ^20^, ^21^, ^22^ Barriers to accessing substance use interventions exist across multiple ecological levels (intrapersonal, interpersonal, organizational, community, and systemic).^22^^,^^23^ Intrapersonal barriers may include cultural beliefs to “tough it out,” self-reliance, and stigma.^23^ Interpersonal factors include a lack of trust in providers in light of historical racism^24^^,^^25^ and parental barriers such as lack of transportation^26^^,^^27^ and fear of being blamed for their child’s problems.^25^ Organizational barriers include an increased likelihood of being discharged or terminated from treatment.^28^ Community-level factors include negative media representations of Black people who misuse substances,^29^ which can alienate Black adolescents and their families, foster stigma and secrecy, and preclude seeking care.^24^^,^^25^ Systemic factors include disparities in access to insurance^22^ and structural racism,^30^^,^^31^ which impacts several factors (eg, employment and financial capital) that limit the ability to afford intervention services.

Black adolescents also have distinct risk and protective factors for substance use.^6^, ^7^, ^8^, ^9^^,^^32^ Racism influences initiation, progression, and polysubstance use among Black adolescents.^33^, ^34^, ^35^, ^36^, ^37^ Black adolescents who believe negative stereotypes about Black people are more likely to misuse substances compared with Black adolescents who do not believe such stereotypes.^38^ Authoritarian parenting, a risk factor for substance misuse in White adolescents, is of lesser risk for Black adolescents.^39^ A strong sense of ethnic-racial identity and parental racial socialization—the process by which Black parents support their children in navigating race-based discriminatory experiences—are protective against substance use among Black adolescents.^38^^,^^40^, ^41^, ^42^, ^43^, ^44^, ^45^

Given existing distinct risk/protective factors for substance use and unique barriers in access to care experienced by Black adolescents, there is a critical need to design and implement substance use interventions that are culturally tailored to the needs of Black adolescents. Cultural tailoring is “the process of developing culturally sensitive interventions.”^46^^(p273)^^,^^47^ Resnicow *et al.* define cultural sensitivity as “the extent to which ethnic/cultural characteristics, experiences, norms, values, behavioral patterns, and beliefs of a target population as well as relevant historical, environmental, and social forces are incorporated in the design, delivery, and evaluation of targeted health promotion materials and programs.”^46^^(p272)^ Cultural tailoring moves beyond targeting,^47^ that is, testing an evidence-based intervention specifically among Black adolescents without any adaptation, and is relevant to all stages of intervention planning from needs assessment to implementation.^48^ Culturally tailored interventions include those that are culturally adapted (ie, initially designed for one population but modified to address the unique needs of a different population) and those that are culturally grounded (ie, initially designed/conceptualized for a specific population).^46^^,^^49^^,^^50^ Cultural tailoring may occur at a surface level or a deep level. Surface-level tailoring seeks to create intervention content and procedures that align with the observable characteristics of the target population. It includes using “people, places, language, music, foods, brand names, locations and clothing preferred by the target audience.”^46^^(p271)^ Deep-level tailoring seeks to integrate the multilevel ecological factors (ie, specific risk and protective factors) that predispose persons to a specific behavior within a target population. It also involves understanding how cultural, historical, and environmental factors have uniquely predisposed the target population and how “the target population perceives the cause, course, and determinants of a particular behavior.”^46^^(p274)^

Prior meta-analyses have demonstrated the efficacy of culturally tailored interventions for substance use prevention among Black adolescents with effect sizes (ESs) ranging from 0.2 to 0.57.^49^^,^^51^, ^52^, ^53^, ^54^ However, none of the previous meta-analyses focused on digital interventions. Prior systematic reviews and meta-analyses examining digital interventions for adolescent substance use have demonstrated efficacy in preventing and reducing substance use^55^, ^56^, ^57^ with small to moderate ESs. However, existing systematic reviews and meta-analyses examining whether the response to the digital interventions differed across racial-ethnic groups are lacking. In fact, a recent scoping review that examined digital interventions for substance use among Black, Hispanic, and American Indian or Alaskan Native youth and adults concluded that digital interventions that attend to cultural sensitivity among these populations are lacking.^58^ Existing systematic reviews examining cultural sensitivity in digital interventions have not focused exclusively on Black adolescents.^59^

Over the past decade, adolescents are increasingly using technology, and more recent data show that more Black teens have access to mobile phones and use social media platforms compared with White and Hispanic teens.^60^^,^^61^ Interventions delivered digitally through various technology media (eg, internet, serious video games, telehealth) have increased access to substance use treatments.^62^^,^^63^ In addition to including content that addresses unique risk/protective factors for the target population, digital interventions offer the ability to feasibly address the needs of adolescents who use substances by reducing barriers to accessing standard in-person care. For example, help-seeking adolescents using a digital intervention may not be limited by reliance on their parent/guardian for transportation.^62^^,^^64^ Digital interventions have additional benefits, such as increased anonymity, inherently reducing the challenges of stigma^65^, and show promise for enhanced intervention engagement (eg, interventions in the form of serious video games are enjoyable for youth).^66^ Implementing digital interventions reduces the burden on primary care providers and trained professionals to provide standard in-person interventions and potentially increases cost savings.^55^^,^^67^ Using digital interventions to prevent substance use problems among youth, specifically youth facing disparities, may reduce barriers to access to care and reduce the burden of the growing mental health crisis.

Cultural tailoring in digital interventions differs in several ways from tailoring that may occur in interventions delivered in person. Because the content for digital interventions is usually the same for all recipients, there may be less human variation in intervention delivery. Fidelity to digital interventions is typically achieved by the delivery of standardized content. Culturally tailored digital interventions can use a variety of music representing the target population, language preferred by the target population, and avatars representing the target group—factors that may be less accessible/practical for interventions delivered in person. These factors necessitate examining the efficacy of culturally tailored digital interventions. However, to our knowledge, no prior systematic review has been conducted specifically examining the efficacy of culturally tailored digital interventions for substance use prevention among Black adolescents. Given the rise in the use of digital interventions as well as the continued need to tailor substance use prevention interventions to address the unique needs of Black adolescents, it is critical to examine the efficacy of these types of interventions.

### Purpose of Review

The goal of this review is to systematically examine the evidence for culturally tailored digital interventions for preventing substance use among Black adolescents.

### Objectives

This systematic review addresses the following questions:

1. What culturally tailored digital substance use prevention interventions exist for Black adolescents?
  a. What are the characteristics of these digital interventions?
  b. What is the level of cultural tailoring in these interventions?
  c. Are these family-based or individual-targeted interventions?
2. Are culturally adapted digital substance use prevention interventions for Black adolescents effective?

## Method

We registered the protocol for this review with PROSPERO (https://www.crd.york.ac.uk/PROSPERO/view/CRD42023452522). The reporting of this systematic review was guided by the standards of the PRISMA statement.^68^

### Search Strategy

An experienced medical librarian (M.C.F.) consulted on methodology and ran a medical subject heading (MeSH) analysis of known key articles provided by the research team using the Yale MeSH Analyzer.^69^ In each database, we ran scoping searches and used an iterative process to translate and refine the searches. For comprehensiveness, the formal search used controlled vocabulary terms and synonymous key words to capture the concepts of technology-based interventions, substance use, and Black adolescents. The search strategy was peer reviewed by a second librarian, who was not otherwise associated with the project, using the Peer Review of Electronic Search Strategies (PRESS) standard.^70^

On August 9, 2023, January 5, 2024, and July 1, 2025, M.C.F. performed a comprehensive search of multiple databases, including Ovid MEDLINE ALL, Embase (Ovid), PsycInfo (Ovid), Cochrane, and Web of Science Core Collection. Both English-language and non-English–language articles were eligible for inclusion. No date limits were applied. All search strategies are in Supplement 1, available online.

### Selection Criteria

We used the PICOS (Population, Intervention, Comparator, Outcome, Study design) framework^71^ in selecting studies to be included.

#### Population

Studies that included Black adolescents ages 12 to 17 years were selected. To maximize the number of interventions retrieved, we included studies with adolescents younger than 12 if the study also included Black adolescents 12 or older. We also included studies that had adolescent populations that were less than 100% Black if the study was testing a digital intervention that included cultural tailoring for Black adolescents.

#### Intervention

Culturally tailored digital interventions for substance use prevention were selected. The intervention description had to include specific ways (surface or deep) in which cultural tailoring occurred specifically for Black adolescents. Interventions tested among Black adolescent populations without any description of cultural tailoring of the specific intervention were excluded. Digital interventions included interventions that were self-administered and delivered primarily through a technology device (eg, text-based, mobile application–based, computer-based, phone-based interventions). Interventions that were delivered by a provider were excluded. In addition, digital interventions could be universal, selective, or indicated interventions.^72^ Universal interventions target the entire population regardless of the existing risk level of the participants. Selective interventions target participants at high risk for the target outcome (eg, high risk for substance use). Indicated interventions target populations already misusing substances or have problems associated with substance use. For interventions that evaluated a culturally tailored digital intervention, we also assessed if tailoring occurred in the delivery of the intervention (implementation).

#### Comparator

Comparators were nondigital interventions (including interventions delivered in person) and no intervention/placebo.

#### Outcome

Outcomes included antecedents of substance use (perception of risk of harm, intentions to misuse substances, self-efficacy to refuse substances); delay in onset of substance use; or prevention, reduction, or avoidance of substance use escalation or worsening. Outcomes of substance use could be measured by self-report or objective report.

#### Study Design

Randomized controlled trials (RCTs) that included assessment of a culturally tailored digital intervention were selected. Although we initially included formative (qualitative needs assessment and usability/acceptability) studies in our registered protocol, we decided to exclude them as it was challenging to synthesize results across all studies. For example, data collected in RCTs (eg, substance use outcomes) were not collected in formative studies.

### Screening Process

Covidence^73^ was used to screen imported articles, extract data from included articles, and assess the risk of bias. Each of 3 authors (D.P., J.M., and U.I.) independently screened all titles, abstracts, and full-text articles in Covidence under the guidance of the lead author (K.A.). D.P., J.M., and U.I. independently completed data extraction, and conflicts at each stage were resolved by consensus and discussion with K.A. We exported extracted data into Microsoft Excel (Microsoft Corporation) for further synthesis. Inter-rater reliability ranged from 79% to 96% for title and abstract screening and 77% to 95% for full-text screening. Two authors independently assessed each article at all stages. No artificial intelligence tools were used at any stage of the screening, data extraction, bias assessment, or synthesis.

### Data Extraction

All data extraction was done using information available in the published article. We did not obtain additional intervention materials from authors of included publications. We extracted data on the characteristics of the included studies (study setting, sample size, demographic characteristics, type of control group, primary outcome and other substance use, and individual antecedents to substance use outcomes).

#### Study Characteristics

The type of control group was coded as either active or passive. Active control describes study arms that received an intervention (eg, therapy-delivery intervention). Passive control describes study arms that received no intervention or received only brochures.

#### Intervention Characteristics

The type of intervention was coded as universal, selective, or indicated. We also determined if the intervention was delivered in a single session or multiple sessions, the setting in which the intervention was delivered (eg, school, clinic), the mode of delivery (eg, computer, mobile phone), and the target population. We also extracted data on the description of cultural tailoring provided in the article and determined the level of cultural tailoring (surface vs deep). Specifically, we looked for statements or words that described tailoring for the Black adolescent population (eg, cultural, tailored for Black youth). Based on the description provided, we determined if the intervention involved surface- or deep-level tailoring. Finally, we extracted information describing the content of the intervention and underlying theory as presented in the article.

#### Outcomes

We extracted the substances and antecedents to substance use (eg, self-efficacy, intention to use a substance) targeted by each intervention and identified the primary outcome in each study. We then determined if the effect of each intervention on these outcomes was significant. We computed ESs for all significant outcomes (Table 1). ESs were either extracted directly from the article (when provided) or computed with provided means, proportions, *F* values, or β coefficients. The mean or proportion change in outcomes was compared between intervention and control groups to compute Cohen’s *d* ESs.^74^ An online calculator was used to compute ESs from means or proportions^75^ and to convert *F* values and β coefficients to Cohen’s *d* ESs.^76^

**Table 1:** Characteristics of Included Studies by Level of Cultural Tailoring

Study: First author, year	Design: Study type, intervention groups,^a^ outcome time points	Sample characteristics: N,^b^ mean age, y (or grade) at baseline, sex, race	Intervention characteristics				Outcomes	
			Intervention name, delivery mode, no. of sessions, intervention type, setting	Targeted determinants	Cultural tailoring (content: surface and/or deep implementation)	Targeted population, TS, PO	Retention rate,^c^ NIH quality rating	Findings from participants in digital intervention group relative to control
**Studies that tested digital interventions with surface-level cultural tailoring**								
Cunningham, 2009^77^^,^^d^	RCT, 3-arm (intervention, active and passive control), post-test and 3 mo	N = 533, age = 16.7, 58% female, 55% Black	SafERteens, computer, single, indicated, ED	Conflict resolution skills, self-efficacy to refuse substances, perceived risk, goal setting, motivation to change	Surface: culturally relevant for urban youth, implementation tailoring: NR	Adolescent only, TS: alcohol, PO: improvement in favorable attitudes toward alcohol use, self-efficacy to refuse alcohol, and readiness to change	81%, 11	Computer intervention was effective in improving alcohol use attitudes at post-test (ES = 0.14, *p* < .001) and at 3 mo (ES = 0.39, *p* < .001); effective in improving self-efficacy to refuse alcohol at post-test (ES = 0.11, *p* < .05), but not at 3 mo; no effect on readiness to change at either post-test or 3 mo
Cunningham, 2010, 2012^78^^,^^79^	RCT, 3-arm (intervention, active and passive control), 3, 6, and 12 mo	N = 726, age = 16.8, 56.5% female, 55.9% Black	SafERteens, computer, single, indicated, ED	Conflict resolution skills, self-efficacy to refuse substances, perceived risk, goal setting, motivation to change	Surface: culturally relevant for urban youth, implementation tailoring: NR	Adolescent only, TS: alcohol, PO: reduction in alcohol misuse	3 mo: 86%, 6 mo: 86%, 12 mo: 84%, 12, 11	Computer intervention was not effective at reducing alcohol use frequency at 3, 6, or 12 mo; not effective at reducing alcohol-related consequences at 6 or 12 mo
Schinke, 2004, 2006, 2010^80^^,^^81^^,^^82^; Schwinn, 2010^83^	RCT, 3-arm (intervention, active and passive control), post-intervention, 1, 2, and 3 y, 4 y, 6 y, and 7 y	N = 514, age = 11.5, 51.4% female, 54% Black	SODAS City Adventure, CD-ROM, multiple (10 and up to 5 annual boosters by 7 y), universal, community setting	Coping skills/self-regulation, refusal skills, perceived norms, self-efficacy, problem solving, decision making	Surface: animated characters with similar racial and ethnic backgrounds, implementation tailoring: NR	Adolescent only, TS: alcohol, PO: past 30-day substance use	3 y: 91%, 4 y: 92%, 6 y: 80%, 7 y: 80%, 9, 11, 11, 11	Computer (adolescent-only arm) intervention improved family involvement at 3 y (ES = 0.16, *p* < .05), but not at 4 y; intervention improved youth decision-making skills at 4 y (ES = 0.33, *p* < .05); no significant effect on problem-solving skills at 4 y; intervention improved peer influences (likelihood of having friends who drink and peer pressure to drink) at post-test, 1, 2, 3, and 7 y (ES = 0.12-1.07, *p* < .05), but not at 4 or 6 y; intervention reduced intentions to drink alcohol in the future at 7 y (ES = 0.34, *p* < .05); intervention improved self-efficacy to refuse alcohol (ES = 0.13-0.34, *p* < .05) at 6 and 7 y; intervention reduced past-month frequency of alcohol use at all points (ES = 0.002-1.5, *p* < .05); intervention reduced past-month frequency of cannabis use at 1, 2, 3, and 4 y (ES = 0.11-1.57, *p* < .05), but not at post-test, 6 y, or 7 y; intervention reduced past-month frequency of cigarette use at all time points (ES = 0.002-0.64, *p* < .05) except post-test; computer intervention reduced alcohol-related consequences (ES = 0.25, *p* < .05) at 6 y
Sussman, 1995 (study 1)^84^	RCT, 2-arm (intervention, active control), post-intervention	N = 267, age = 12.75, 50% female, 41% Black	Rap, videotape, single, universal, school	Perceived peer norms	Surface: rap music, hip-hop, clothing, slang, and dancing, urban street setting with graffiti, implementation tailoring: African American adults collected data	Adolescent only, TS: tobacco, PO: intention to smoke in the future	NR, 6	Videotape intervention had no effect on future intention to smoke at post-test; the culturally tailored intervention was perceived by participants to be more accurate in its depiction of the African American culture (ES = 0.32, *p* < .01) and way of life (ES = 0.33, *p* < .05); participants who viewed the culturally tailored intervention were more likely than participants in the control group to report that they had learned a great deal (ES = 0.28, *p* < .05), that the intervention was helpful (ES = 0.37, *p* < .01); compared with adolescents from other racial/ethnic groups, Black adolescents were more likely to report that the intervention was helpful (ES = 0.55, *p* < .001) and that the intervention was reflective of African American culture (ES = 0.28, *p* < .05)
Walton, 2014^85^	RCT, 3-arm (intervention, active and passive control), 3, 6, and 12 mo	N = 714, age = 14.9, 57% female, 63.7% Black	Project Chill, computer, single, universal, clinic (primary care)	Self-efficacy, perception of risk, intention to use, personal responsibility, perceived norms, and coping skills	Surface: cultural relevance in language (in key messages and scripts), item listed checkboxes (reasons to avoid using drugs), and scenario topics, implementation tailoring: NR	Adolescent only, TS: marijuana, PO: initiation and frequency of cannabis use	88%, 13	Computer intervention improved perceived risk from cannabis use (ES = 0.29, *p* < .05) and self-efficacy to refuse cannabis use at post-test (ES = 0.35, *p* < .05); no effect was found on intentions to use cannabis; computer intervention showed reduction in cumulative use of cannabis over past 12 mo (ES = 0.18, *p* < .05), cannabis use frequency at 3 mo (ES = 0.12, *p* < .05) and 6 mo (ES = .12, *p* < .05), but not at 12 mo; other drug use frequency at 3mo (ES = 0.09, *p* < .05), but not at 6 or 12 mo; no effect on alcohol use outcomes
**Studies that tested digital interventions with deep-level cultural tailoring**								
Schinke, 2011^67^	RCT, 2-arm (intervention, passive control), post-intervention	N = 546, age = 12.75, 100% female, 65.2% Black	SODAS, gender specific for minority girls and their mothers, CD-ROM, multiple (10), selective, home/online	Parent–adolescent relationship; parental monitoring, parent–adolescent communication; parental rules/expectations about substance use; self-efficacy for managing stress; coping skills, self-regulation; self-efficacy to refuse peer pressure; knowledge about substances, self-esteem	Deep: intervention content on impact of racism, implementation tailoring: NR	Adolescent and parent dyads, TS: alcohol, marijuana, tobacco, other illicit substances, PO: past 30-day substance use	95%, 10	Online intervention improved mother–daughter communication (ES = 0.16, *p* < .05), family rules about drugs (ES = .32, *p* < .0001), parental monitoring (ES = 0.29, *p* < .01), normative beliefs (ES = 0.29, *p* < .001), depression (ES = 0.17, *p* < .05), self-efficacy to refuse drugs (ES = 0.22, *p* < .001), and intention to use drugs (ES = 0.12, *p* < .05); online intervention reduced frequency of alcohol use (ES = 0.2, *p* < .01); no effect was found for body esteem, cigarettes, marijuana, and prescription drugs
**Studies that tested digital interventions with surface and deep-level cultural tailoring**								
Murry, 2019^86^	RCT, 3-arm (intervention, active and passive control), post-intervention and 23 mo	N = 421, age = NR (6th graders), 54% female, 100% Black	Pathways for African American Success (PAAS), computer, multiple (6), universal, community setting	Parent–adolescent relationship, parent–adolescent communication; communication about substance use and rules, adaptive racial socialization, intention to use substances, risk resistance skills, future orientation, and peer affiliations	Surface and deep: avatars representing the target population, focus on racial socialization, and dealing with racism, implementation tailoring: known community leader contacted families, African American community members provided feedback on assessment measures, collected data and helped troubleshoot any technological difficulties	Adolescent and parent dyads, TS: alcohol, tobacco, marijuana, cocaine, hallucinogens, methamphetamines, heroin, huffing, ecstasy, or prescription drugs, PO: reduction in substance use, intention	81%, 10	Technology intervention improved parenting in relation to challenging topics (rules about substance use, sexual communication, and racial socialization) (ES = 0.77, *p* < .01) and reduced intention to engage in risk behaviors (ES = 0.24, *p* < .05); technology intervention did not significantly influence supportive parenting or affiliation with deviant peers; technology intervention reduced risk for substance use and sexual risk behaviors (ES = 0.35, *p* < .05)^e^

Note: ED = emergency department; ES = effect size; NIH = National Institutes of Health; NR = not reported; PO = primary outcomes for this systematic review; RCT = randomized controlled trial; TS = targeted substances.

a Passive vs active: active control includes a control arm that received a nondigital intervention; passive control groups received no intervention/education materials.

b Entire study sample.

c Overall retention rate in the study at each time point reported or at the latest time point.

d Same intervention and population as Cunningham, 2010 and 2012, studies, but includes only a subset of the sample to examine antecedents, as enrollment was still ongoing for the overall study.

e Measures of sexual and substance use risk were combined.

### Risk of Bias Assessment

Three authors (D.P., J.M., U.I.) independently assessed the quality of individual studies. The lead author (K.A.) resolved differences in quality ratings. The National Institutes of Health (NIH) tool for quality assessment of controlled intervention studies^87^ was used to assess the risk of bias among RCTs. The NIH quality assessment tool consists of 14 questions that assess the following sources of bias: adequacy of randomization, allocation concealment, blinding, similarity of groups at baseline, dropout rate, similarity of treatment across study arms, sample size, and intention-to-treat analysis. Each criterion has 5 options (yes, no, not reported, not applicable, and cannot determine). Each criterion was given a value of 1 for a yes response and 0 for all other responses with a possible range of 0 to 14. As used in prior studies,^55^^,^^88^ we characterized study quality using the following rubric: poor (<7), fair (7-9), and good (≥10).

### Synthesis Method

We conducted a narrative synthesis summarizing results by study characteristics, intervention characteristics, and study outcomes/findings. We initially planned to conduct a formal meta-analysis to compare ES of self-reported 30-day substance use, stratified by the level of cultural tailoring, but this was not possible due to the limited number of studies (1 study reported only deep-level tailoring, and 1 study reported a combination of surface- and deep-level tailoring), heterogeneity in the assessment measures used, variable assessment time points, sample population (adolescent only vs dyads), and the content/level of cultural tailoring.

## Results

### Study Selection

**Figure 1:**
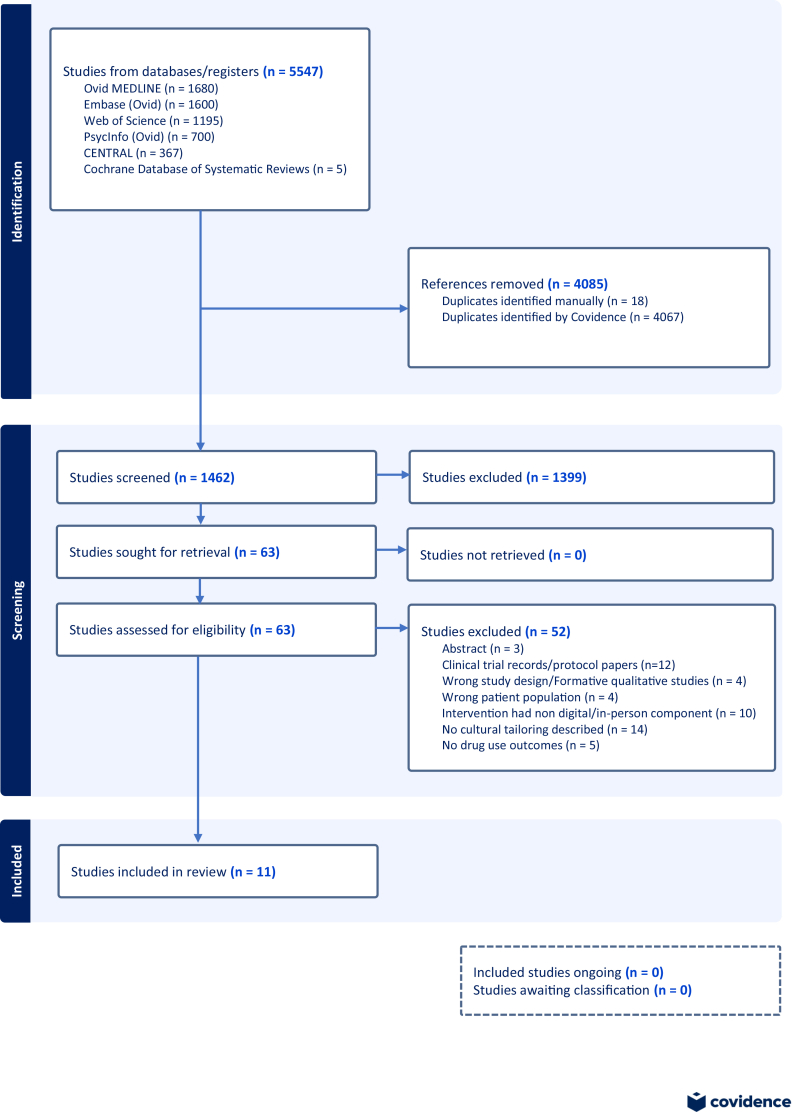
PRISMA Flow Diagram Delineating Study Selection Process

The final search retrieved 5,547 references, which were pooled in EndNote 21^89^ and then uploaded to Covidence^73^ for screening. In Covidence, duplicates were identified and removed, leaving 1,462 references for screening. A PRISMA flow diagram is presented in Figure 1.

### General Study Characteristics

Our inclusion criteria were met by 11 articles comprising 6 separate studies, each of which tested a different intervention for a total of 6 different interventions (Table 1).^67^^,^^77^, ^78^, ^79^, ^80^, ^81^, ^82^, ^83^, ^84^, ^85^, ^86^ Two of the 6 studies included different articles evaluating the same interventions at different time points. For example, 1 of the 6 interventions was included in 3 articles with 3-, 6-, and 12-month outcomes,^77^, ^78^, ^79^ and another intervention was included in 4 articles with outcomes of 1 to 3 years, 4 years, 6- years, and 7 years.^80^, ^81^, ^82^, ^83^ Four studies included outcomes with ≥12 months of follow-up. Included studies comprised 3,188 adolescents (range 267-726/study), with the proportion of Black adolescents ranging from 41% to 100% (n = 109-421). One study included an adolescent population that was entirely Black.^86^ The mean age of participants at the time of study entry was 13.6 years, with a range of 11.5 to 16.7, and 61.5% of participants were female (including 1 study that had 100% female participants). All studies were RCTs, and 5 of these interventions reported evidence-based theories that informed the intervention (Table 1; Supplement 2, available online).^67^^,^^77^, ^78^, ^79^, ^80^, ^81^, ^82^, ^83^, ^85^, ^86^^,^^85^^,^^86^

Cunningham *et al.*^77^, ^78^, ^79^ targeted adolescents ages 14 to 18 years in an urban emergency department setting using a computer-delivered, single-session intervention and reported past-year aggression and alcohol use outcomes. Strengths of this study include the demonstrated feasibility of testing a digital intervention in an emergency department setting and the follow-up time of up to 1 year. This intervention showed efficacy for targeting antecedents to substance use (eg, alcohol-related attitudes), but not for actual substance use. Limitations include the limited description of cultural tailoring.

Schinke and Schwinn and colleagues^80^, ^81^, ^82^, ^83^ tested a multisession (10 sessions plus booster sessions) universal intervention that targeted adolescents ages 10 to 12 years. Similar to the study by Cunningham *et al.*,^77^, ^78^, ^79^ description of cultural tailoring was minimal. Strengths include the long-term follow-up (up to 7 years after intervention) with demonstrated sustained intervention effects on alcohol use and cigarette use at all time points and cannabis use up to 4 years after intervention (Table 1). Inclusion of booster sessions likely contributed to the sustained effects across time.^80^, ^81^, ^82^, ^83^ Limitations include the lack of consistent reporting of the antecedents to substance use across all time points.

Sussman *et al.*^84^ also tested a single-session intervention for smoking prevention that targeted adolescents ages 11 to 13 years in urban settings. Strengths include the description of specific ways cultural tailoring was done and the assessment of the effect of cultural tailoring on intervention outcomes. Limitations include the lack of description of the theory that informed intervention development, the lack of long-term follow-up, and the lack of assessment of actual substance use. The intervention was not found to be effective at impacting intention to smoke after intervention.

Walton *et al.*^85^ tested a single-session intervention aimed at prevention of cannabis use that targeted adolescents ages 12 to 18 years without prior use of cannabis. Strengths include demonstrated feasibility of delivering a digital intervention in the primary care setting, follow-up of participants up to 1 year, demonstrated effects on antecedents to substance use (eg, self-efficacy to refuse cannabis), and cannabis use.^85^ Limitations include the minimal description of cultural tailoring.

Schinke *et al.*^67^ tested a gender-specific intervention, tailored for Black and Hispanic girls, that targeted both adolescent girls and their parents in urban settings. Cultural tailoring included content that addressed racism. The intervention demonstrated efficacy on antecedents (eg, mother–daughter communication, self-efficacy) as well as on alcohol use after test. However, no long-term follow-up data are available.

Murry *et al.*^86^ targeted Black adolescents ages 11 to 12 years and their caregivers in rural settings. Intervention had an impact on antecedents to substance use (eg, rules about substance use) and substance use (alcohol, cigarettes, marijuana, prescription drugs, and other illicit drugs). The study included 100% Black adolescents. Other strengths include detailed description of cultural tailoring that spans both intervention content and implementation and long-term follow-up as long as 22 months after intervention. A limitation was that the substance use outcome was combined with sexual risk outcomes such that it was not possible to determine the specific effect on substance use.

### Digital Intervention Characteristics

Included interventions were self-administered interventions designed to target adolescents only (4 studies)^78^^,^^80^^,^^84^^,^^85^ or adolescents and their parents (2 studies).^67^^,^^86^ Substances targeted by these interventions included alcohol (5 studies),^67^^,^^78^^,^^80^^,^^85^^,^^86^ marijuana (4 studies),^67^^,^^80^^,^^85^^,^^86^ tobacco (4 studies),^67^^,^^80^^,^^84^^,^^86^ and other illicit substances (3 studies).^67^^,^^80^^,^^86^ These interventions were delivered using a CD-ROM/computer (5 studies)^67^^,^^78^^,^^80^^,^^85^^,^^86^ or videotape (1 study).^84^ Interventions were designed to be completed in a single session (3 studies)^77^, ^78^, ^79^^,^^84^^,^^85^ or multiple sessions (3 studies).^67^^,^^80^, ^81^, ^82^, ^83^^,^^86^ Interventions with multiple sessions ranged from 6 to 10 sessions. Most interventions were universal (4 studies), with 1 selective intervention^67^ and 1 indicated intervention.^77^, ^78^, ^79^ The settings where these interventions were tested included community (2 studies),^80^^,^^86^ home (1 study),^67^ school (1 study),^84^ emergency department (1 study),^77^, ^78^, ^79^ and primary care (1 study).^85^ Digital intervention methods used included videos employing role plays (1 study)^84^ and interactive animated applications with avatar(s) that included role play, tutorials, quizzes, or drill-and-practice exercises (5 studies).^67^^,^^78^^,^^80^^,^^85^^,^^86^

### Targeted Determinants

Intentions to use substances in the future (5 studies) and self-efficacy (4 studies) were the most frequently targeted determinants among interventions. Self-efficacy includes confidence in one’s ability to refuse substances, resist peer pressure, and manage internal distress. Other individual-level determinants included coping skills, perceived peer norms, knowledge about the effects of substances, affiliation with peers with high-risk behaviors, problem solving, and decision making.

Interventions that included parents also frequently targeted the parent–adolescent relationship, parent–adolescent communication, and parental expectations/rules regarding substance use. One study that targeted both parents and adolescents targeted adaptive racial socialization.^86^

### Cultural Tailoring

All studies described cultural tailoring at the content level (eg, having animated characters that represent the Black population (surface-level content tailoring), including content that addresses specific protective factors such as racial socialization (deep-level content tailoring), whereas 2 studies described tailoring at the implementation level (eg, having community members representative of the Black population contact participants and conduct assessments).^84^^,^^86^ Most interventions (4 studies) reported only surface-level content tailoring (eg, depicting animated characters, virtual communities representative of the target population). One intervention reported deep-level content adaptations that targeted a specific risk factor (racism),^67^ whereas 1 study reported both surface- and deep-level adaptations (animated characters representative of the Black population and adaptive racial socialization).^86^ Interestingly, the 2 interventions that included deep-level tailoring also targeted both adolescents and their parents,^67^^,^^86^ whereas all the surface-level interventions targeted only adolescents.^78^, ^80^, ^84^, ^85^ In this sense, targeting parents and teens could be construed as being part of deep tailoring, in recognition of the important role that parents have in shaping outcomes of Black adolescents. Overall, we found differences in the content of cultural tailoring across all studies, with some lacking details/examples of the specific cultural tailoring enacted (Supplement 2, available online).

Most studies tested whether the given culturally tailored intervention was effective by comparing it with an active and/or passive control group. However, only the study by Sussman *et al.*^84^ specifically examined the effect of cultural tailoring on study outcomes by comparing the tailored intervention with the same intervention devoid of cultural tailoring.

### Outcomes

#### Substance Use Outcomes

Five interventions assessed substance use outcomes (Table 1). Four of them reported significant findings for reduced substance use (alcohol, marijuana, tobacco, and other illicit substance use) with ESs including 0.002 to 1.5 for alcohol use, 0.11 to 1.57 for marijuana use, 0.002 to 0.64 for tobacco use, and 0.009 to 0.35 for other drugs (Table 1). Of these 5 interventions, 3 were multisession interventions,^67^^,^^80^, ^81^, ^82^, ^83^^,^^86^ and 2 were single-session interventions.^77^, ^78^, ^79^^,^^85^ All the multisession interventions reported significant improvements in substance use, whereas one^85^ of 2 single-session interventions reported significant improvements. Furthermore, of these 5 interventions, 3 were universal,^80^, ^81^, ^82^, ^83^^,^^85^, ^86^ 1 was selective,^67^ and 1 was indicated.^77^, ^78^, ^79^ Universal and selective interventions reported significant effects in improving substance use, whereas the indicated intervention did not. Of the 2 single-session interventions, one was a universal intervention that yielded significant reductions in cannabis use,^85^ whereas the other was an indicated intervention that had no effect on alcohol use.^78^^,^^79^ Of the 4 interventions that investigated substance use outcomes ≥12 months after intervention, 1 reported no significant findings at 12 months,^79^ and the other 3 reported significant findings persisting at 12 months,^85^ 23 months,^86^ and up to 7 years.^81^, ^82^, ^83^

#### Antecedents to Substance Use

Of 6 studies, 5 reported improvements in antecedents to substance use (Table 1). Included interventions demonstrated a positive impact on self-efficacy to refuse substances with ESs ranging from 0.11 to 0.35, intentions to use substances in the future with ESs ranging from 0.12 to 0.34, perceived risk of harm from marijuana (ES = 0.29), attitudes toward alcohol use (ES = 0.14), decision-making skills (ES = 0.33), peer influences (likelihood of having peers who drink and peer pressure to drink [ESs = 0.12-1.07]), normative beliefs about drugs (ES = 0.29), and alcohol-related consequences (ES = 0.25). One study measured readiness to change but did not report significant findings. Two studies assessed parenting outcomes. One of these studies demonstrated effect on family involvement (ES = 0.16), mother–daughter communication (ES = 0.16), family rules about drugs (ES = 0.32), and parental monitoring (ES = 0.29), whereas the other study showed effect on parenting communication about sensitive topics (rules about substance use, sexual communication, and racial socialization) with an ES of 0.77.

#### Impact of Cultural Tailoring

Sussman *et al.*^84^ found that compared with adolescents of other racial/ethnic groups, Black adolescents were more likely to rate the culturally tailored intervention as helpful (ES = 0.55) and were also more likely to report that the intervention was reflective of African American culture (ES = 0.28). However, the culturally tailored intervention did not differ from the nontailored intervention on substance use–related outcomes, ie, effect on intention to use tobacco.

#### Retention Rate

Five studies reported study retention rates. The overall study retention rates at the latest outcome time point ranged from 80% to 95%. The mean retention rates for the digital intervention arm, active control arm, and passive control arm were 87.8%, 83.3%, and 87.2%, respectively.

### Quality Assessment

Five studies were of fair to good quality, and 1 study was of low quality (Supplement 3, available online). Areas of strength across all studies include adequacy of randomization, adherence to study protocols, similar treatments across study groups outside of the intervention, using valid and reliable measures, and prespecifying outcomes. Areas contributing to the low-quality rating included blinding and reporting whether the sample size was adequate, allocation concealment, and intent-to-treat analysis.

## Discussion

The distinct risks for substance use that affect Black adolescents and the unique protective factors that they and their families possess highlight the need for culturally tailored interventions. The barriers in accessing care that Black adolescents experience suggest a role for culturally tailored services that could include culturally tailored digital interventions. This systematic review examined the effects of existing culturally tailored digital interventions for substance use prevention among Black adolescents. Overall, 4 of 6 interventions reported significant effects for reducing substance use, and 5 of the 6 interventions reported significant findings for improving antecedents to substance use. ESs ranged from small to large for alcohol and marijuana, small to moderate for tobacco and other drugs, and small to medium for antecedents to substance use outcomes. The impact of cultural tailoring was examined in only 1 of 5 studies, and there was a wide variation in cultural tailoring across the studies included.

Consistent with prior systematic reviews/meta-analyses examining the efficacy of culturally tailored nondigital interventions for substance use prevention among Black adolescents,^49^^,^^51^^,^^52^^,^^54^ 4 studies in this review found significant effects on substance use with similar ESs to those reported for nondigital interventions.^49^^,^^51^, ^52^, ^53^, ^54^ However, without specifically testing the impact of cultural tailoring, it is challenging to draw conclusions about the overall effect of cultural tailoring or the impact of surface-level vs deep-level tailoring on observed intervention effects. Our review found only 2 interventions that described deep-level tailoring. It would seem that given existing differences in the patterns of substance use, risk/protective factors, and consequences of substance use, there would be a need for not only surface-level tailoring (eg, use of animated characters depicting target population), but also deep-level tailoring (eg, addressing specific risk and protective factors of the Black population).

It is noteworthy, however, that in other studies testing digital interventions without specific cultural tailoring found these to be effective for impacting tobacco use outcomes among Black adolescents.^90^^,^^91^ Pasick *et al.*^47^ suggested that the benefits of cultural tailoring may lie more with tailoring the implementation (eg, having members of the target population involved in delivering the intervention, delivering the intervention in communal spaces that resonate with the target population) than the intervention itself, although both are needed. Two of the studies reviewed^84^^,^^86^ describe such implementation factors: one study found significant effects on improving substance use risk,^86^ whereas the other, assessed as low quality, found no effects on intention to use.^84^ The implementation factors described included having community leaders contact families and having members of the Black community administer interviews/questionnaires and help troubleshoot any technical difficulties with setting up the digital intervention. Future studies are needed to examine these effects to inform the development, adaptation, and implementation of culturally tailored digital interventions among Black adolescents.

Questions left unanswered include what outcomes are impacted by cultural tailoring, what mechanisms account for observed impacts, and what aspects of implementation need to be tailored. For example, is cultural tailoring in digital interventions necessary for engagement and retention, or does it directly impact substance use outcomes, or does engagement/retention mediate the impact of tailoring on substance use outcomes? In this systematic review, given the small number of studies overall and the variability in levels of cultural tailoring, we could not conduct a quantitative analysis to determine pooled ESs for the interventions overall or stratified by variation in cultural tailoring. To determine the impact of cultural tailoring, it will be important to isolate this effect from other effects that might include location of intervention delivery, number of sessions, and type of intervention.

We found a wide variation in the description and integration of cultural tailoring among the studies. Some studies simply described using avatars/characters that resembled the Black population,^78^ whereas 1 study was culturally grounded (ie, initially designed/conceptualized for a specific population).^86^ This wide variation suggests a lack of consensus about the core components of cultural tailoring needed for substance use prevention using digital interventions among Black youth, as well as the limited research completed to date on the effectiveness of cultural tailoring for substance use prevention among Black adolescents. Future directions may include a taxonomy of cultural tailoring amenable to digital translation and delivery that can form the basis for further systematic study.

Of the 5 studies that reported substance use outcomes,^67^^,^^78^, ^79^, ^80^, ^81^, ^82^, ^83^^,^^86^ all of which were of good quality, 4 reported significant reductions in substance use with small to large ESs. Our results also suggest that digital interventions delivered in multiple sessions may be more effective in impacting substance use than digital interventions delivered in single sessions. Prior meta-analyses evaluating the efficacy of digital interventions for adolescent substance use prevention and treatment found that digital interventions with multiple sessions may be more effective compared with those with single sessions.^55^^,^^92^ Although caution is necessary in interpreting these findings given the limited number of studies, this finding suggests that providing repeated opportunities to practice skills may allow adolescents and/or parents to incorporate these skills into habitual practice and is consistent with studies showing that repeated practice builds strong habits that can cue a person into action regardless of attitudes/intentions.^93^ Multisession interventions may address more relevant risk/protective factors than is possible in a single session. Furthermore, all the universal digital interventions yielded significant improvements in substance use^80^, ^81^, ^82^, ^83^^,^^85^^,^^86^ compared with the indicated intervention,^79^ which showed no effect on substance use. The indicated intervention was also delivered in a single session making it challenging to isolate impact of dose vs level of risk of substance use. Given the limited number of indicated interventions in this review, conclusions cannot be drawn on the efficacy of universal vs indicated digital interventions. Future studies are needed to elucidate the effect of digital interventions on Black adolescent substance use by intensity of the intervention (single session vs multisession), type of intervention (universal, selective, or indicated), depth of cultural adaptation, type of substance use, and severity of substance use among adolescents to inform implementation efforts.

Of the 6 studies, 5 reported significant improvements in antecedents to substance use (intentions, self-efficacy, and refusal skills).^67^^,^^79^, ^80^, ^81^, ^82^, ^83^^,^^85^^,^^86^ Most interventions targeted intentions to use substances and self-efficacy, including self-efficacy to resist peer pressure and refuse substances and self-efficacy to use coping skills. Intentions to use substances and self-efficacy are established antecedents to substance use among adolescents in general^94^^,^^95^ and among Black adolescents specifically.^95^, ^96^, ^97^, ^98^ Similarly, interventions that involved parents targeted determinants relevant to all adolescents, such as parent–adolescent communication and supportive relationships. Improving self-efficacy and parent–adolescent communication may also lend themselves to digital translation, as adolescents can watch avatars model refusal skills while engaging in game-based tasks to improve self-efficacy and communication skills. These findings highlight the importance of targeting known risk factors for substance use for all adolescents and raise the question of what aspects of evidence-based interventions need to be tailored for Black adolescents. Prior reviews have documented mixed findings regarding the efficacy of nontailored interventions for substance use prevention among Black adolescents,^99^ suggesting the need for future research to further clarify aspects of tailoring that are salient for substance use prevention among Black adolescents.

Five of the 6 studies^67^^,^^77^, ^78^, ^79^, ^80^, ^81^, ^82^, ^83^, ^85^, ^86^ documented retention rates upwards of three-fourths of the enrolled population, suggesting the feasibility of conducting substance use prevention research using digital interventions among Black adolescents. Additionally, despite the limited number of studies, these interventions were tested in various settings (home/online, school, primary care, and community). Considering the unique barriers in access to care that Black adolescents experience, these findings underscore the need to examine further factors that foster engagement and retention with digital interventions across various settings to inform future implementation efforts that allow for the scalability of these interventions among this historically underserved group.

We found a limited number of studies during this review, which is consistent with studies with other health targets documenting low prevalence of culturally tailored digital interventions for racial/ethnic minority populations.^59^^,^^100^ We also found low variation in the types of digital delivery formats, with 5 interventions using computers/CD-ROMs for intervention delivery. As the number of Black adolescents who have and use mobile phones increases, it will be important to develop and test culturally tailored interventions that employ other forms of digital technology (video games, virtual reality, extended reality, mobile applications, text-based, web-based). Understanding the impact of these newer forms of digital interventions among Black adolescents will extend our understanding of the field while ensuring equitable access to interventions with these newer digital formats.

In only 1 study, the entire study sample identified as Black.^86^ Drawing conclusions about the efficacy of culturally tailored digital interventions for substance use prevention among Black adolescents is limited by studies that were not powered to determine impact on Black adolescents specifically. Only 1 study described a reason for recruiting adolescents from racial and ethnic groups other than Black: “to test for ethnic differences in reactions to the tailored intervention.”^84^ This was also the only study to assess the impact of cultural tailoring specifically. Future studies testing culturally tailored interventions for Black adolescents should provide a rationale for the study sample being recruited to allow for substantive conclusions on the effect of cultural tailoring on Black adolescents.

Some limitations should be noted. As discussed, wide variation in the content of cultural tailoring and variation in the proportion of Black adolescents who enrolled in the studies limit conclusions about the efficacy of cultural tailoring for substance use prevention among Black adolescents. The limited number of studies overall, which was even more limited by the lack shared substance use outcome measures, precluded a meta-analysis overall and by subgroups. Most studies in this review did not report or assess tailoring relevant to implementation strategies, an important aspect of tailoring that may impact outcomes. Most of the interventions in this review were computer-based, limiting the generalizability of these findings to newer digital intervention formats. As we did not obtain additional intervention materials, this review is limited by the reliance of cultural tailoring described in published articles.

In conclusion, this systematic review provides an initial examination of available culturally tailored digital interventions for substance use prevention among Black adolescents and an evaluation of the efficacy of these interventions. Overall, findings suggest that culturally tailored digital interventions can be helpful for Black adolescents, although the wide variation in culturally tailored content, limited types of digital interventions, and heterogeneity of the studies and sample population warrant further examination for definitive conclusions. Given the rapid advances in digital technologies and the pressing need to curtail the rise in substance use among Black adolescents, it is imperative to design, test, and implement digital interventions that meet the needs of Black adolescents. The most important conclusion that we can draw from this study is that there is a serious dearth of research into this important area of substance use prevention. To advance the field, important areas of future research include developing a taxonomy of cultural tailoring for digital interventions to guide both the reporting and the assessment of these interventions and comparative trials that examine the effect of cultural tailoring on efficacy and implementation outcomes to identify effective tailored components that would in turn inform both the design and the implementation of these interventions.

## CRediT authorship contribution statement

**Kammarauche Aneni:** Writing – review & editing, Writing – original draft, Supervision, Methodology, Investigation, Funding acquisition, Formal analysis, Data curation, Conceptualization. **Destiny Pegram:** Writing – review & editing, Writing – original draft, Methodology, Data curation. **Jenny Meyer:** Writing – review & editing, Writing – original draft, Methodology, Data curation. **Melissa C. Funaro:** Writing – review & editing, Writing – original draft, Software, Methodology, Data curation. **Roberta L. Bruhn:** Writing – review & editing, Methodology, Formal analysis. **Uzochukwu Imo:** Writing – review & editing, Data curation. **Aaron Hogue:** Writing – review & editing, Supervision. **José Szapocznik:** Writing – review & editing, Supervision, Methodology. **Ijeoma Opara:** Writing – review & editing, Supervision, Methodology, Investigation.
