# Supplementary material for: Systematic Review: Culturally Tailored Digital Substance Use Prevention Interventions for Black Adolescents
Source: JAACAP Open. 2026 Apr 2;4(4):569–88. doi: 10.1016/j.jaacop.2026.03.007 (PMC13420601; doi:10.1016/j.jaacop.2026.03.007)
Supplement: Supplementary Data [file mmc1.docx]

Supplement 1: All search strategies

Ovid MEDLINE(R) ALL

| 1 | child/ or adolescent/ or (child* or adolescen* or teen or teenager* or youth*).tw,kf. |
| --- | --- |
| 2 | exp Black people/ or "Black or African American"/ or (Black* or African American* or Afro*).tw,kf. |
| 3 | Minority Groups/ or ((underrepresented or under represented or racial or ethnic) adj5 (minority or minorities)).tw,kf. |
| 4 | urban/ or urban.tw,kf. |
| 5 | 2 or 3 or 4 |
| 6 | exp Substance-Related Disorders/ or (addict* or ((alcohol or alcoholism or alcoholic* or amphetamine* or "angel dust" or benzodiazepine* or cannabis or cigarette* or chemical or cocaine or drinking or drug* or e-cig* or hashish or heroin or inhalant* or marihuana or marijuana or methamphetamine* or morphine or nicotine or narcotic or opioid* or opium or opiate* or phencyclidine or substance* or stimulant* or smoking or tobacco or vaping) adj2 ("use" or misuse or abuse))).tw,kf. |
| 7 | exp wearable electronic devices/ or Computers, Handheld/ or Smartphone/ or electronic mail/ or mobile applications/ or digital technology/ or internet based intervention/ or exp cell phone/ or telemedicine/ or text messaging/ or video games/ or (app based or cell phone* or cellular phone* or computer* or digital or e health or ehealth or email* or electronic tool or fitness tracker* or handheld* or hand held* or internet-based or ipad* or iphone* or mobile or mhealth or m health or mhealth or smart glass* or smartphone* or smart phone* or tablet* or technology or technologies or telemedicine or tele medicine or telenursing or tele nursing or telehealth or tele health or telecare or tele care or text messag* or text-based or text delivered or videogam* or video gam* or web app* or web based or wearable* or ((online or electronic or app or apps or application* or web or website* or computer) adj2 (intervention or program*))).tw,kf. |
| 8 | 1 and 5 and 6 and 7 |
| 9 | video recording/ or videodisc recording/ or videotape recording/ or video*.tw,kf. |
| 10 | 1 and 5 and 6 and 9 |
| 11 | 8 or 10 |

Embase (Ovid)

| 1 | Child/ or Adolescent/ or (child* or adolescen* or teen or teenager* or youth*).tw,kw. |
| --- | --- |
| 2 | exp Black person/ or (Black* or African American* or Afro*).tw,kw. |
| 3 | minority group/ or ((underrepresented or under represented or racial or ethnic) adj5 (minority or minorities)).tw,kw. |
| 4 | Urban area/ or Urban population/ or urban.tw,kw. |
| 5 | 2 or 3 or 4 |
| 6 | exp drug dependence/ or (addict* or ((alcohol or alcoholism or alcoholic* or amphetamine* or "angel dust" or benzodiazepine* or cannabis or cigarette* or chemical or cocaine or drinking or drug* or e-cig* or hashish or heroin or inhalant* or marihuana or marijuana or methamphetamine* or morphine or nicotine or narcotic or opioid* or opium or opiate* or phencyclidine or substance* or stimulant* or smoking or tobacco or vaping) adj2 ("use" or misuse or abuse))).tw,kf. |
| 7 | web-based intervention/ or personal digital assistant/ or exp mobile phone/ or e-mail/ or exp mobile application/ or digital technology/ or telemedicine/ or text messaging/ or exp video game/ or exp wearable computer/ or exp personal computer/ or (app based or cell phone* or cellular phone* or computer* or digital or e health or ehealth or email* or electronic tool or fitness tracker* or handheld* or hand held* or internet-based or ipad* or iphone* or mobile or mhealth or m health or mhealth or smart glass* or smartphone* or smart phone* or tablet* or technology or technologies or telemedicine or tele medicine or telenursing or tele nursing or telehealth or tele health or telecare or tele care or text messag* or text-based or text delivered or videogam* or video gam* or web app* or web based or wearable* or ((online or electronic or app or apps or application* or web or website* or computer) adj2 (intervention or program*))).tw,kf. |
| 8 | 1 and 5 and 6 and 7 |
| 9 | limit 8 to conference abstracts |
| 10 | 8 not 9 |
| 11 | videorecording/ or video*.tw,kf. |
| 12 | 1 and 5 and 6 and 11 |
| 13 | limit 12 to conference abstracts |
| 14 | 12 not 13 |
| 15 | 10 or 14 |

APA PsycInfo (Ovid)

| 1 | Adolescent Health/ or (child* or adolescen* or teen or teenager* or youth*).tw. |
| --- | --- |
| 2 | Blacks/ or African cultural groups/ or "people of color"/ or (Black* or African American* or Afro*).tw. |
| 3 | Minority Groups/ or ((underrepresented or under represented or racial or ethnic) adj5 (minority or minorities)).tw. |
| 4 | Urban Environments/ or urban*.tw. |
| 5 | 2 or 3 or 4 |
| 6 | exp "Substance Use Disorder"/ or "substance use prevention"/ or (addict* or ((alcohol or alcoholism or alcoholic* or amphetamine* or "angel dust" or benzodiazepine* or cannabis or cigarette* or chemical or cocaine or drinking or drug* or e-cig* or hashish or heroin or inhalant* or marihuana or marijuana or methamphetamine* or morphine or nicotine or narcotic or opioid* or opium or opiate* or phencyclidine or substance* or stimulant* or smoking or tobacco or vaping) adj2 ("use" or misuse or abuse))).tw. |
| 7 | exp Mobile Technology/ or text messaging/ or exp mobile devices/ or mobile health/ or mobile applications/ or digital technology/ or computer games/ or digital gaming.mp. or digital interventions/ or exp Computer Assisted Therapy/ or (app based or cell phone* or cellular phone* or computer* or digital or e health or ehealth or email* or electronic tool or fitness tracker* or handheld* or hand held* or internet-based or ipad* or iphone* or mobile or mhealth or m health or mhealth or smart glass* or smartphone* or smart phone* or tablet* or technology or technologies or telemedicine or tele medicine or telenursing or tele nursing or telehealth or tele health or telecare or tele care or text messag* or text-based or text delivered or videogam* or video gam* or web app* or web based or wearable* or ((online or electronic or app or apps or application* or web or website* or computer) adj2 (intervention or program*))).tw. |
| 8 | 1 and 5 and 6 and 7 |
| 9 | video-based interventions/ or Digital Video/ or video*.tw. |
| 10 | 1 and 5 and 6 and 9 |
| 11 | 8 or 10 |

Web of Science Core Collection - Advanced Search (Clarivate)

Core Collection includes: Science Citation Index Expanded (SCI-EXPANDED), Social Sciences Citation Index (SSCI), Arts & Humanities Citation Index (A&HCI), Conference Proceedings Citation Index – Science (CPCI-S), Conference Proceedings Citation Index – Social Science & Humanities (CPCI-SSH), Book Citation Index – Science (BKCI-S), Book Citation Index – Social Sciences & Humanities (BKCI-SSH), Emerging Sources Citation Index (ESCI), Current Chemical Reactions (CCR-EXPANDED)

| 5 | #4 AND #3 AND #2 AND #1 |
| --- | --- |
| 4 | TS=(video* or "app based" or "cell phone*" or "cellular phone*" or computer* or digital or "e health" or ehealth or email* or "electronic tool*" or "fitness tracker*" or handheld* or "hand held*" or "internet based" or ipad* or iphone* or mobile or mhealth or "m health" or mhealth or "smart glass*" or smartphone* or "smart phone*" or tablet* or technology or technologies or telemedicine or "tele medicine" or telenursing or "tele nursing" or telehealth or "tele health" or telecare or "tele care" or "text messag*" or "text based" or "text delivered" or videogam* or "video gam*" or "web app*" or "web based" or wearable*) OR TS=((online or electronic or app or apps or application* or web or website* or computer) NEAR/2 (intervention or program*)) |
| 3 | TS=((addict* or ((alcohol or alcoholism or alcoholic* or amphetamine* or "angel dust" or benzodiazepine* or cannabis or cigarette* or chemical or cocaine or drinking or drug* or e-cig* or hashish or heroin or inhalant* or marihuana or marijuana or methamphetamine* or morphine or nicotine or narcotic or opioid* or opium or opiate* or phencyclidine or substance* or stimulant* or smoking or tobacco or vaping) NEAR/2 ("use" or misuse or abuse)))) |
| 2 | TS=(Black* or "African American*" or Afro*) OR TS=((underrepresented or "under represented" or racial or ethnic) NEAR/5 (minority or minorities)) OR TS=urban |
| 1 | TS=(child* or adolescen* or teen or teenager* or youth*) |

Cochrane Database of Systematic Reviews, Cochrane Central Register of Controlled Trials (Wiley)

| #1 | (child* or adolescen* or teen or teenager* or youth*):ti,ab,kw |
| --- | --- |
| #2 | (Black* or (African NEXT American*) or Afro*):ti,ab,kw OR ((underrepresented or "under NEXT represented" or racial or ethnic) NEAR/5 (minority or minorities)):ti,ab,kw OR (urban):ti,ab,kw |
| #3 | (addict*):ti,ab,kw OR ((alcohol or alcoholism or alcoholic* or amphetamine* or "angel dust" or benzodiazepine* or cannabis or cigarette* or chemical or cocaine or drinking or drug* or e-cig* or hashish or heroin or inhalant* or marihuana or marijuana or methamphetamine* or morphine or nicotine or narcotic or opioid* or opium or opiate* or phencyclidine or substance* or stimulant* or smoking or tobacco or vaping) NEAR/2 ("use" or misuse or abuse)):ti,ab,kw |
| #4 | (video* or "app based" or (cell NEXT phone*) or (cellular NEXT phone*) or computer* or digital or "e health" or ehealth or email* or (electronic NEXT tool*) or (fitness NEXT tracker*) or handheld* or (hand NEXT held*) or "internet based" or ipad* or iphone* or mobile or mhealth or "m health" or mhealth or (smart NEXT glass*) or smartphone* or (smart NEXT phone*) or tablet* or technology or technologies or telemedicine or "tele medicine" or telenursing or "tele nursing" or telehealth or "tele health" or telecare or "tele care" or (text NEXT messag*) or "text based" or "text delivered" or videogam* or (video NEXT gam*) or (web NEXT app*) or "web based" or wearable*):ti,ab,kw OR (((online or electronic or app or apps or application OR applications or web or website or websites or computer) NEAR/2 (intervention or program or programs or programming))):ti,ab,kw |
| #5 | #1 AND #2 AND #3 AND #4 |
