# Supplementary material for: Systematic Review: Culturally Tailored Digital Substance Use Prevention Interventions for Black Adolescents
Source: JAACAP Open. 2026 Apr 2;4(4):569–88. doi: 10.1016/j.jaacop.2026.03.007 (PMC13420601; doi:10.1016/j.jaacop.2026.03.007)
Supplement: Supplementary Data [file mmc2.docx]

Supplement 2. Detailed Summaries of Digital Interventions Included in this Systematic Review

| Author and Year | Description of the program | Noteworthy points (strengths and limitations) |
| --- | --- | --- |
| Cunningham et al. (2009, 2010, 2012) | Adolescents aged 14-18 years old presenting to the ED in Flint Michigan and reporting both past year aggression and alcohol consumption in the past 12 months were eligible to participate.  Enrolled participants were randomized into one of three groups: computer session, counselor session or brochure.  The computer intervention was a one session intervention which was informed by principles of Motivational Interviewing and included normative feedback and skill building exercises. Content focused on goals, personalized feedback about alcohol, violence, weapon carriage, decisional balance exercises for potential benefit from staying away from drinking and fighting, tailored role plays on anger management, conflict resolution, alcohol refusal and avoiding drinking and driving, referral. The computer intervention involved a buddy that guided participants through the above intervention components and provided audio feedback when the participant made appropriate choices.  Description of cultural tailoring was minimal. Authors describe making intervention culturally relevant for inner city youth with Black youth making 50% of the study population.  Outcomes were assessed at baseline, post-test, 3 months, 6 months and 1 year. Antecedent outcomes included alcohol use attitudes, readiness to change, and self-efficacy. Substance use outcomes included past-year alcohol use frequency, binge drinking, alcohol consequences, past year cigarette and illicit drug use.  The computer session lasted on average 29 minutes (median) | Demonstrated feasibility in an ED setting, theory informed intervention, participants followed up to year. Demonstrated efficacy on antecedents. However, no effects on eventual substance use were found. |
| Schinke et al.  (2004, 2006, 2010)  Schwinn et al. (2010) | Adolescents aged 10-12 years involved in community centers in New York City offering after-school programs were eligible to participate.  Enrolled participants were enrolled into one of three groups: CD-ROM intervention, CD-ROM + parent intervention and control.  The CD-ROM intervention was a ten-session intervention informed by the family interaction theory, social learning theory and problem behavior theory. The content included goal setting, coping, decision making, effective communication, and time management. The intervention included both instruction and guided skill practice activities. During each session, youth encountered realistic obstacles that they had to maneuver by employing a problem-solving strategy (Stop, Options, Decide, Act, Self-Praise). Intervention also included a yearly booster.  Description of cultural tailoring was minimal. Characters in the computer intervention depicted similarities to the youth’s ethnic-racial background.    Outcomes were assessed at baseline, post-test, 1 year, 2 years, 3 years, 4 years, 6 years and 7 years. Antecedent outcomes included family involvement, peer influences (number of peers who use drugs, perception of influence peers have on their drug use, ability to refuse peer influences), problem solving, refusal skills/self-efficacy to refuse. Substance use outcomes included past-month use of alcohol, cigarettes, marijuana and other drugs, past-month alcohol related consequences  Each computer session lasted 45 minutes | Intervention with the longest follow-up (up to 7 years) with sustained effects both on antecedents and substance use. Intervention is theory informed. However, different outcomes were assessed at different time points (besides the drug use outcomes which were measured consistently across time). |
| Sussman et al. (1995) | Adolescents aged 11-13 years old in urban school settings in southern, eastern or central sections of Los Angeles County were eligible to participate.  Enrolled participants were assigned to one of two groups at the school level: culturally tailored video intervention and a non-culturally tailored version of the same intervention.  The video intervention was delivered in one session and designed to motivate youth to refuse cigarettes based on peer disapproval. Participation involved viewing a videotape which describes a storyline in which an adolescent being rejected by a peer due to cigarette use.  Cultural tailoring involved depicting setting, language, and music reflective of Black youth.  Outcomes were assessed at post-test. Antecedent outcomes include intention to smoke in the future. No actual substance use was measured. Outcomes related to cultural tailoring were also assessed and included likelihood that the video depicted African American way of life and culture, perception of learning, perception that the video was helpful. | Directly assessed the impact of cultural tailoring on study outcomes. However, there was no long-term follow-up and no assessment of actual substance use. |
| Walton et al. (2014) | Adolescents aged 12-18 years in urban primary care clinics in the Midwest who reported no life-time cannabis use were eligible to participate.  Enrolled participants were assigned to computer brief intervention, therapist brief intervention and control  One session intervention informed by principles of Motivational Interviewing. Content included goals and values, normative feedback about drug use, deciding reasons to avoid using drugs, interactive role play scenarios guided by a computer character that engages and motivates the participant to make choices that improves the computer character, summary to reinforce learning.  Cultural tailoring reflected feedback from predominantly African American youth and included tailoring language, and scenario topics  Outcomes were assessed at baseline, posttest, 3 months, 6 months and 12 months. Urine drug test was done for each time point. Antecedent outcomes included perceived risk of occasional and regular cannabis use, self-efficacy to refuse cannabis, intention to use cannabis. Substance use outcomes included past-3 month use and frequency of using cannabis, past-3-month frequency, quantity and heavy drinking of alcohol, past-3-month frequency of using illicit and non-medical prescription drugs  Computer session lasted on average 33 minutes | Strengths include demonstrated feasibility of delivering a digital intervention in the primary care setting, follow-up of participants up to one year, demonstrated effects on antecedents to substance use (e.g., self-efficacy to refuse cannabis) and cannabis use. Limitation includes the minimal description of cultural tailoring. |
| Schinke et al. (2011) | Black and Hispanic adolescent girls aged 10-13 years and their mothers were recruited online and through newspaper adverts in the New York, New Jersey and Connecticut areas.  Enrolled dyads were randomized to one of 2 conditions: a computer/online-delivered gender-specific intervention or control (no intervention).  Computer/Online intervention consists of ten sessions of gender-specific intervention for ethnic minority girls informed by family interaction theory. Content included mother-daughter communication, mother-daughter closeness, assertiveness, praise, parental self-efficacy and parent monitoring, substance use education, media influences. The intervention also focused on skill training including conflict management, stress and mood management, coping skills and strategies, problem solving using the Stop, Options, Decide, Action, Self-Praise (SODAS), drug refusal skills, adolescent self-efficacy, and dealing with racism. Both mother and daughter accessed the online intervention with separate logins but had exercises that they had to complete with each other.  Cultural tailoring involved including content about racism and dealing with racism.  Outcomes were assessed at baseline and post-test. Antecedent outcomes include parent-child communication, family rules about drug use, parental monitoring, perception of peer drug use (normative beliefs), depression, body esteem, self-efficacy to refuse drugs and intentions to use. Substance use outcomes included past-month use of cigarettes, alcohol, marijuana and prescription drugs. | Strengths include specific tailoring for Black and Hispanic girls. The intervention was also informed by family interaction theory and cultural tailoring included content that addressed racism. The intervention demonstrated efficacy on antecedents (mother-daughter communication, family rules, parent monitoring, intentions to use drugs and self-efficacy) as well as on alcohol use at post-test. However, no long-term follow-ups are reported. |
| Murry et al. (2019) | Black adolescents aged 11-12 years and their caregivers living in rural counties in Western Tennessee and spoke English were eligible for the study. Rural counties had to meet specific criteria: rurality index scores >11, >30% African American residents, >600 African American teens in the targeted age range, teen pregnancy rates of 69% (higher than average for Tennessee), negative overall health indicators.  Enrolled dyads were randomized to one of three conditions: technology format, group format, literature control (mailed education material). Parents and youth accessed the technology intervention separately and then had a joint dyadic session. Technology sessions occurred at specific community settings with computers provided.  Technology intervention consists of six sessions informed by the self-efficacy and control theory, established understanding of the role that parenting, racial identity and self-esteem can mitigate risk among Black youth. Intervention was designed to target both sexual risk and drug use risk. Content included parent-child communication, rules about risk behaviors, parental monitoring and discipline, family values, parental racial socialization, resisting peer pressure, future orientation, and dealing with racism  Cultural tailoring occurred at the intervention and implementation level. Intervention characters represented the population communities and intervention content addressed racism, ethnic identity, and parental racial socialization. Implementation level tailoring included refining measures and procedures with feedback from Black community members, having community members troubleshoot technological challenges.  Outcomes were assessed at baseline, posttest and 22 months after posttest. Antecedent outcomes included supportive parent-youth relationship, adaptive racial socialization, parent-child communication, communication about rules/expectations about drug use, intention to engage in risk behaviors, affiliation with peers with deviant behaviors. A latent variable, challenging topics, represent rules about drug use risk, sexual communication and racial socialization, while another latent variable, supportive parenting represented parental support, open communication and frequency of communication about substances. A Substance use outcomes include past use of substances (cigarettes, alcohol, marijuana, cocaine, hallucinogens, methamphetamines, heroin, huffing, ecstasy, prescription drugs).  Each technology session lasts 45 minutes. | Strengths include a detailed description of cultural tailoring. Tailoring encompassed both intervention components and implementation processes. Study included only Black adolescents. However, actual substance use outcomes were combined with sexual risk outcomes in reporting of results. Technology intervention improved parenting skills, reduced intention to engage in risk behaviors and reduced substance use and sexual risk behaviors. |
