# Supplementary material for: Systematic Review: Culturally Tailored Digital Substance Use Prevention Interventions for Black Adolescents
Source: JAACAP Open. 2026 Apr 2;4(4):569–88. doi: 10.1016/j.jaacop.2026.03.007 (PMC13420601; doi:10.1016/j.jaacop.2026.03.007)
Supplement: Supplementary Data [file mmc4.pdf]

[redacted] a systematic review

To enable PROSPERO to focus on COVID-19 submissions, this registration record has undergone basic automated checks for eligibility and is published exactly as submitted. PROSPERO has never provided peer review, and usual checking by the PROSPERO team does not endorse content. Therefore, automatically published records should be treated as any other PROSPERO registration. Further detail is provided here.

Review methods were amended after registration. Please see the revision notes and previous versions for detail.

Citation

[redacted]

Review question

1. What culturally adapted digital substance use prevention interventions are available for black adolescents? What are the characteristics of these digital interventions? What age groups have been targeted?

2. What are the settings in which the interventions take place?

Searches

1. Ovid MEDLINE, Embase (Ovid), APA PsycINFO (Ovid), Web of Science, and Cochrane

Types of study to be included

randomized controlled trials, formative/development papers

Condition or domain being studied

Substance use

Participants/population

Black adolescents

Intervention(s), exposure(s)

Digital/tech-based interventions: web-based, computer-based, text-based, telehealth, app-based, videogames

Comparator(s)/control

in-person intervention, non-digital intervention

Main outcome(s)

prevalence of digital interventions for substance use prevention among Black adolescents;

frequency of substance use

Additional outcome(s)

cultural adaptation of digital interventions for Black adolescents

Data extraction (selection and coding) [1 change]

1. Authors and year of publication

2. Title of study

3. Country of study

4. Aim of study

5. Sample size

6. Study design

7. Population age, school grade, race, sex

8. Population description

9. Exclusion criteria

10. Type of substance targeted

11. Theoretical framework that informed intervention

12. Name of digital/technology intervention

13. Type of technology platform: web-based, mobile application,

14. Intervention setting

15. Duration of intervention

16. Dosage (total minutes)

17. Booster sessions

18. Description of intervention

19. Type of intervention: universal, selective, indicated

20. Target of intervention: adolescent, parents, parents and adolescents

21. Follow-up duration

22. Comparison group

23. Type of control group: active or passive

24. Primary outcome: antecedents of substance use (perception of risk of harm, intentions to misuse, self-efficacy to refuse), delay in onset of substance misuse/ prevention of escalation of substance misuse among those that do not meet criteria for substance use disorder

25. Other outcomes targeted in included studies

26. Content themes of digital/technology-based intervention

27. Effect size and statistical significance

28. Retention rate

29. Level of cultural adaptation: surface or deep

Risk of bias (quality) assessment

Two authors will independently review selected studies to assess for the risk of bias using the NIH Quality Assessment of Controlled Intervention Studies. Risk of bias will be assessed at the individual study level. Each study will be rated as Good, Fair or Poor. Any discrepancies identified between authors will be resolved by discussion between authors.

Strategy for data synthesis

1. A qualitative synthesis will be undertaken to characterize available culturally adapted interventions digital interventions for substance use. We will synthesize our findings by the type of intervention (universal, selective, indicated).

2. Criteria under which data will be quantitatively synthesized: We will synthesize interventions using the value of the effect size reported in the studies if we have a sufficient number of studies to compute effect size by the study primary outcome (substance use or antecedents of substance use). If studies are sufficiently homogenous, a meta-analysis will be conducted. .

Analysis of subgroups or subsets

If there are enough articles, we will examine outcomes of culturally adapted interventions to non-adapted interventions to examine their efficacy.

Contact details for further information

[redacted]

Organisational affiliation of the review

[redacted]

Review team members and their organisational affiliations [1 change]

[redacted]

[redacted]

Type and method of review

Systematic review

Anticipated or actual start date

28 August 2023

Anticipated completion date [1 change]

30 June 2024

Funding sources/sponsors

[redacted]

Grant number(s)

State the funder, grant or award number and the date of award

[redacted]

Conflicts of interest

Language

English

Country

United States of America

Stage of review

Review Ongoing

Subject index terms status

Subject indexing assigned by CRD

Subject index terms

Adaptation, Physiological; Adolescent; Black People; Humans; Substance-Related Disorders

Date of registration in PROSPERO

21 August 2023

Date of first submission

10 August 2023

Stage of review at time of this submission [2 changes]

Stage	Started	Completed
Preliminary searches	Yes	Yes
Piloting of the study selection process	Yes	No
Formal screening of search results against eligibility criteria	Yes	No
Data extraction	Yes	No
Risk of bias (quality) assessment	Yes	No
Data analysis	No	No

Revision note

Updated the projected completion timeline for the project

The record owner confirms that the information they have supplied for this submission is accurate and complete and they understand that deliberate provision of inaccurate information or omission of data may be construed as scientific misconduct.

The record owner confirms that they will update the status of the review when it is completed and will add publication details in due course.

Versions

21 August 2023
21 August 2023
30 October 2023
22 February 2024
